# Transcription factor Wilms’ tumor 1 regulates developmental RNAs through 3′ UTR interaction

**DOI:** 10.1101/gad.291500.116

**Published:** 2017-02-15

**Authors:** Ruthrothaselvi Bharathavikru, Tatiana Dudnakova, Stuart Aitken, Joan Slight, Mara Artibani, Peter Hohenstein, David Tollervey, Nick Hastie

**Affiliations:** 1 Medical Research Council Institute of Genetics and Molecular Medicine, University of Edinburgh, Western General Hospital, Edinburgh EH4 2XU, United Kingdom;; 2 Wellcome Trust Centre for Cell Biology, University of Edinburgh, Edinburgh EH9 3BF, United Kingdom;; 3 Roslin Institute, The University of Edinburgh, Easter Bush, Midlothian EH25 9RG, United Kingdom

**Keywords:** WT1, FLASH, 3′ UTR, hybrids, RNA secondary structures, developmental pathways

## Abstract

Wilms’ tumor 1 (WT1) is essential for the development and homeostasis of multiple mesodermal tissues. Despite evidence for post-transcriptional roles, no endogenous WT1 target RNAs exist. Using RNA immunoprecipitation and UV cross-linking, we show that WT1 binds preferentially to 3′ untranslated regions (UTRs) of developmental targets. These target mRNAs are down-regulated upon WT1 depletion in cell culture and developing kidney mesenchyme. *Wt1* deletion leads to rapid turnover of specific mRNAs. WT1 regulates reporter gene expression through interaction with 3′ UTR-binding sites. Combining experimental and computational analyses, we propose that WT1 influences key developmental and disease processes in part through regulating mRNA turnover.

## Abstract (precis)

Bharathavikru et al. show that Wilms’ tumour 1 (WT1) binds preferentially to 3′ UTRs of developmental targets, which are down-regulated upon WT1 depletion in cell culture and developing kidney mesenchyme. Combining experimental and computational analyses, they propose that WT1 influences key developmental and disease processes in part through regulating mRNA turnover.

The different steps in gene expression, from transcription through a series of post-transcriptional events, are closely interconnected, and some multifunctional proteins regulate several points in this pathway. One potential example is the Wilms’ tumor gene *WT1*. Mice lacking *Wt1* die at mid-gestation through defective coronary vasculature, suffer from congenital diaphragmatic hernia, and have no kidneys, gonads, spleen, or adrenals. All of these defects can be traced to a key role for WT1 in the development of tissues derived from the intermediate and lateral plate mesoderm. In humans, germline *WT1* mutations lead to the eponymous pediatric cancer, genitourinary anomalies, and, in some cases, congenital diaphragmatic hernia and heart disease (Chau and Hastie 2012).

Furthermore, WT1 is a key regulator of the balance between mesenchymal and epithelial states in these tissues, being required for the mesenchyme-to-epithelial transition (MET), a key step in nephron formation, and the epithelial-to-mesenchyme transition (EMT) that produces coronary vascular progenitors from the epicardium. WT1 also plays essential roles in adult tissue homeostasis. Hence, ubiquitous deletion of *Wt1* in adult mice leads to acute kidney glomerulosclerosis, atrophy of the exocrine pancreas and spleen, and widespread reduction in bone and fat (Chau et al. 2011). A recent study showed that WT1 is reactivated during tissue repair and adult tumorigenesis, where it is required in the mesenchymal component and vasculature for tumor growth (Wagner et al. 2014).

The molecular mechanisms by which WT1 fulfils these apparently diverse roles have been attributed to its transcriptional function. Accordingly, there is substantial evidence that WT1 binds genomic DNA and regulates transcription, acting as either an activator or a repressor (Essafi et al. 2011; Toska and Roberts 2014). A growing number of physiological WT1 transcriptional targets have been identified in development, homeostasis, and disease (Motamedi et al. 2014; Dong et al. 2015; Kann et al. 2015). However, the evidence suggests that transcriptional regulation is not the only WT1 function.

The two major essential isoforms (Barbaux et al. 1997; Hammes et al. 2001) conserved throughout vertebrate evolution are created by an alternative splice that inserts three amino acids (lysine–threonine–serine [KTS]) between the third and fourth zinc finger (Hastie 2001). The first suggestion that WT1 may function post-transcriptionally came from Larsson et al. (1995), who showed that the +KTS isoforms associate with splicing factors. WT1 was then shown to integrate into active splice complexes by interacting with the splicing factor U2AF65 (Davies et al. 1998). Moreover, WT1 binds both RNA and DNA with similar binding efficiencies, as demonstrated by structural and kinetic studies (Caricasole et al. 1996; Bardeesy and Pelletier 1998; Zhai et al. 2001). Structural modeling studies also identified an RNA recognition motif (RRM) in WT1 (Kennedy et al. 1996). Further support for post-transcriptional roles for WT1 was provided by Niksic et al. (2004), who showed that all isoforms shuttle between the nucleus and cytoplasm, where they are located on actively translating ribosomes. In particular, the +KTS isoforms were shown to recruit transcripts containing a viral RNA sequence to polysomes, regulating their translation (Bor et al. 2006).

A considerable amount of data therefore support roles for WT1 from splicing through to translation. However, causal evidence for direct WT1 functions in specific steps and understanding of the mechanisms involved have been limited by the absence of characterized endogenous WT1 RNA targets. To address this, we identified WT1-interacting RNAs in mouse embryonic stem (ES) cells and a mesonephric cell line by a combination of RNA immunoprecipitation (RIP) and sequencing (RIP-seq) and a modification of the CLASH (cross-linking ligation and sequencing of hybrids) UV cross-linking technique (Helwak et al. 2013) termed FLASH (formaldehyde-assisted cross-linking and sequencing of hybrids). The latter identifies RNAs that interact directly with WT1 as well as RNA–RNA duplexes formed by these target RNAs. The interaction of intramolecular RNA hybrids is increased in the presence of WT1. This study provides strong evidence that the tumor suppressor protein WT1 regulates physiologically important RNAs and their stability through interactions with 3′ untranslated regions (UTRs).

## Results and Discussion

### WT1 binds to multiple categories of RNA

**Figure 1.**
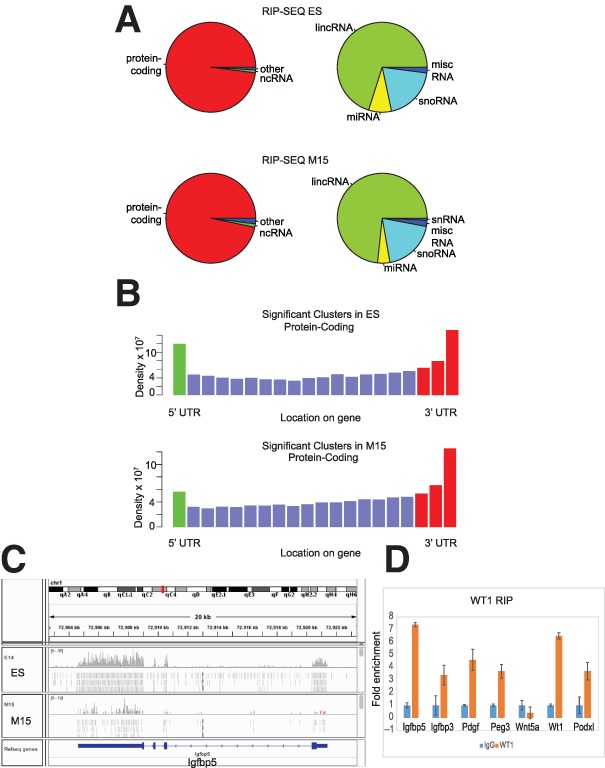
WT1 binds to multiple categories of RNA. (*A*) Pie charts of clusters assigned to protein-coding, noncoding, and other biotypes in ES (*top left*) and M15 (*bottom left*) and the proportion of ncRNAs in ES (*top right*) and M15 (*bottom right*). (*B*) The density of clusters identified by RIP-seq in ES (*top*) and M15 (*bottom* for all protein-coding RNAs (blue bars) and the density at 5′ and 3′ ends (green and red bars, respectively). (*C*) Genome browser snapshot of alignment of RIP-seq reads from ES and M15 cell lines mapping to *Igfbp5*. (*D*) Validation of the WT1-interacting protein-coding RNA biotype. *Igfbp5*, *Igfbp3*, *Pdgf*, *Peg3*, *Wnt5a*, *Wt1*, and *Podxl* interaction was confirmed by RIP in the M15 cell line analyzed by quantitative RT–PCR (qRT–PCR) with target-specific primers.

RIP (Bharathavikru and Dudnakova 2016) was performed on ES cells and mesonephric M15 cells along with negative control RIP to identify RNAs that interact with endogenous WT1. The specificity of the antibody was verified by immuno-pull-down and Western blotting (Supplemental Fig. S1A). Recovered RNAs were identified by RT–PCR and Illumina sequencing. Overall numbers of reads obtained are shown in Supplemental Figure S1B. Reads were mapped to the mouse genome and analyzed for different RNA categories (Fig. 1A). RIP data were processed to identify regions in which the number of uniquely aligned reads passed statistical significance (false discovery rate [FDR] < 0.05; minimum of five reads), and overlapping regions of aligned reads were clustered (minimum of five reads) using pyCRAC software (Webb et al. 2014). The RNA biotypes of these clusters were determined using Ensembl annotations. Strikingly, WT1 targets were predominantly (96%) identified as protein-coding mRNAs (Fig. 1A). Among noncoding RNA (ncRNA) targets, the majority was long intergenic ncRNAs (lincRNAs) followed by microRNAs (miRNAs) and small nucleolar RNAs (snoRNAs). Closer examination of the position of significant clusters on protein-coding genes revealed higher recovery of sequences at the 5′ and 3′ ends of transcripts and the UTRs (Fig. 1B). A number of transcripts had notably high read coverage, including those for *Igfbp5*, shown as an example in Figure 1C.

To confirm the sequence data, RIP reactions were performed followed by PCR across different regions of selected protein-coding transcripts (Fig. 1D; Supplemental Fig. S1C). For most of the selected targets, including *Igfbp5*, the results were in good agreement with the RIP-seq analysis, showing significant enrichment toward the 3′ UTR. In order to assess the functional relevance of the WT1-interacting RNA, a gene ontology (GO) analysis was performed. Several developmental pathways that are regulated by WT1 were identified as significant GO categories in this analysis, including coronary vasculature development and the wnt signaling pathway (Supplemental Fig. S1D,E; Supplemental Tables S1, S2).

### UV cross-linking confirms WT1 enrichment over the 3′ UTR of mRNAs

**Figure 2.**
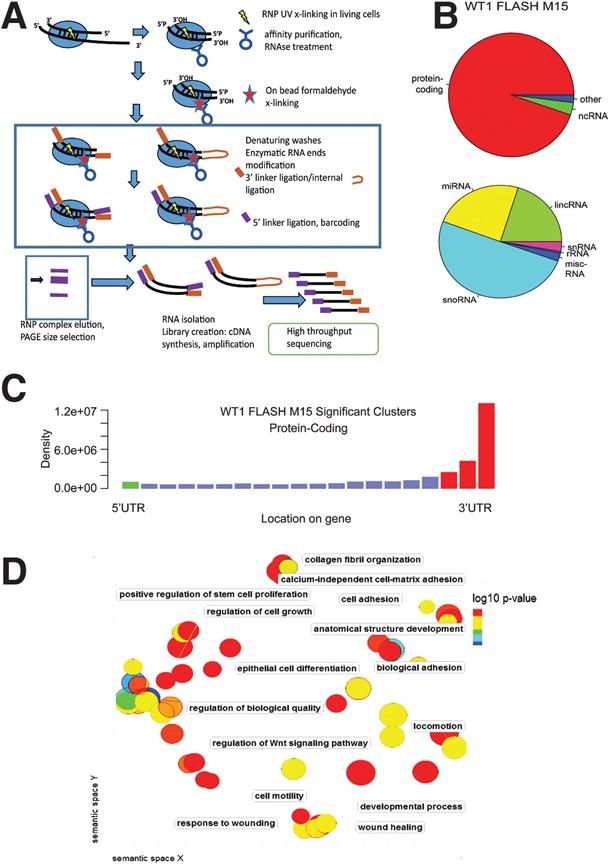
UV cross-linking confirms WT1 enrichment over 3′ UTR of mRNAs. (*A*) Schematic of FLASH protocol. (*B*) Pie charts showing the proportion of clusters of protein-coding, noncoding, and other biotypes (*top*) and ncRNAs (*bottom*) in the FLASH data. (*C*) The density of clusters identified in WT1 FLASH for protein coding (blue bars) and the density at the 5′ and 3′ ends (green and red bars, respectively). (*D*) REVIGO (reduce and visualize GO) plot of *P*-value-based GO terms associated with WT1-interacting RNA identified by FLASH.

To identify specific protein–RNA interaction sites, we adopted a UV cross-linking approach. Since ncRNAs were identified among WT1-interacting RNA (Fig. 1A), we applied an experimental strategy that can identify RNA–RNA interactions. The CLASH technique can identify both RNA–protein and RNA–RNA interactions but requires the use of epitope-tagged “bait” proteins (Helwak et al. 2013). We developed a modified technique, FLASH (Fig. 2A). This allowed the antibody-based immunoprecipitation of endogenous WT1 and the recovery of cross-linked RNAs in the M15 cell line.

Reads obtained from FLASH experiments were analyzed separately for protein–RNA interactions and protein-associated RNA–RNA hybrids (Travis et al. 2014; Webb et al. 2014). The assignment of significant clusters to biotypes showed that the major category of interacting RNA was protein-coding transcripts (Fig. 2B), consistent with the RIP-seq data. The most enriched ncRNAs were snoRNAs (different from RIP-seq due to the difference in methodology) followed by similar numbers of miRNAs and lincRNAs. On mRNAs, WT1-binding sites showed strong enrichment over the 3′ end of the ORFs and 3′ UTRs (Fig. 2C), in comparison with input and control IgG FLASH (Supplemental Fig. S2). GO analysis of the terms associated with the WT1-interacting RNAs (Supplemental Table S3) showed enrichment for developmental pathways, cell adhesion, and cell migration, similar to the RIP-seq data. A GO chart generated by REVIGO (reduce and visualize GO) is shown in Figure 2D. Thus, WT1 was found to interact with developmental regulators, as identified by both RIP-seq and FLASH.

### WT1 associates with RNA at the 3′ UTR through secondary structures

The FLASH analysis includes ligation steps that can lead to the formation of hybrid cDNAs derived from two independent RNA molecules (intermolecular) or noncontiguous sequences of the same RNA (intramolecular/gene-self). To investigate whether WT1 interacts with secondary structured RNA or other RNA duplexes, we analyzed the WT1 FLASH data for chimeric reads derived from RNA hybrids using the hyb software pipeline (Travis et al. 2014). In the FLASH data, 0.49% of reads were identified as hybrids, consistent with recovery in previous analyses. The control input data for the WT1 RIP were similarly analyzed, which showed 0.03% hybrids.

**Figure 3.**
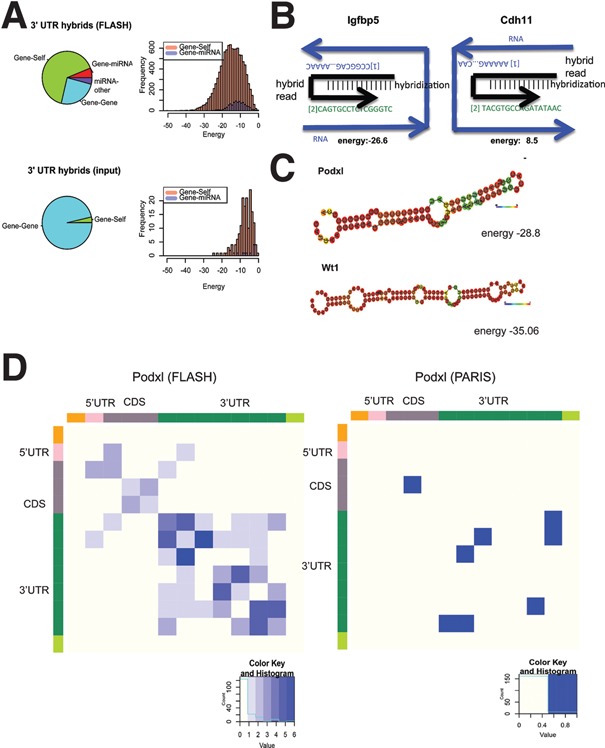
WT1 associates with RNA at the 3′ UTR through secondary structures. (*A*) Pie charts of WT1 FLASH-associated hybrids (*top*) compared with input (*bottom*) in M15 cells analyzed as energy maps. (*B*) Local hybridization in 3′ UTRs for the representative targets *Igfbp5* and *Cdh11*. (*C*) RNA fold predictions of secondary structures of *Podxl* and *Wt1* 3′ UTR interactions. (*D*) Heat map representation of 3′ UTR intramolecular interactions in *Podxl* RNA identified by FLASH and PARIS (psoralen analysis of RNA interactions).

A representation of the different hybrids obtained in WT1 FLASH is shown in Figure 3A. The different data sets of WT1-interacting RNA show a reasonable overlap (60% of the protein-coding genes identified by FLASH were also found by RIP-seq) (Supplemental Table S4). Interactions recovered within 3′ UTR sequences were most frequently intramolecular, comprising 52% of hybrids. Intermolecular interactions between mRNAs and miRNAs were ∼10-fold less frequent, comprising 5% of hybrids with 964 unique interactions. Notably, in human AGO1 data, mRNA–miRNA interactions were threefold more numerous than intramolecular interactions within 3′ UTRs (Helwak et al. 2013). In addition, the mean energy of intermolecular 3′ UTR base-pairing was −15.1 kcal/mol in the FLASH data, significantly less than in the control (−7.3 kcal/mol; *P* < 2.2 × 10^−16^ by Wilcoxon rank sum test) (Fig. 3A; Supplemental Fig. S3A,B). Several interactions were between noncontiguous sequences that were sufficiently close to allow folding prediction for the RNA to assess their stability. Representative examples of local contacts (Fig. 3B) and those with extended secondary structures are shown in Figure 3C. These structures have lower hybridization energy, indicating a more stable structure.

During the preparation of this report, RNA–RNA interactions using psoralen cross-linking were identified. We compared the RNA hybrids identified by PARIS (psoralen analysis of RNA interactions) (Lu et al. 2016) with the RNA hybrids identified by WT1 FLASH. Analysis of WT1-interacting *Podxl*, *Igfbp3*, *Upk3b*, and *Ctdsp2* revealed a difference in the location and energy of the hybrids and the strength of the hybrids (Fig. 3D; Supplemental Fig. S3C). Thus, in the presence of WT1, there is an increase in the number of stable intramolecular RNA interactions, supporting a role for WT1 as a nucleating center for these interactions. Recent studies with the dsRNA-binding protein Staufen (Sugimoto et al. 2015) show secondary RNA structures to be common and functionally important for gene expression. WT1 interaction at the 3′ UTR of targets also shows secondary structures, suggesting a role in regulating RNA stability. To assess the potential of the predicted WT1-bound RNA structures, we analyzed the hybrid sequences for the presence of any miRNA-binding sites. A significant proportion of hybrids was found to harbor clusters of miRNA-binding sites (Supplemental Fig. S3D; Supplemental Table S5).

Since its identification as an RNA-binding protein, it has been speculated that WT1-associated RNA binding may not be determined exclusively by sequence. This was indeed shown with WT1 zinc fingers and RNA aptamers cloned based on the SELEX motifs, which showed that WT1 zinc finger–RNA interaction was not just sequence-dependent but also required a hairpin loop structure adjacent to the consensus motif (Zhai et al. 2001). This is in agreement with our hybrids analysis, which shows that WT1 interacts with stable RNA secondary structures. The exact contribution of sequence and structural requirements for the interaction requires further investigation.

### WT1 regulates the expression of a subset of the RNA-binding targets

**Figure 4.**
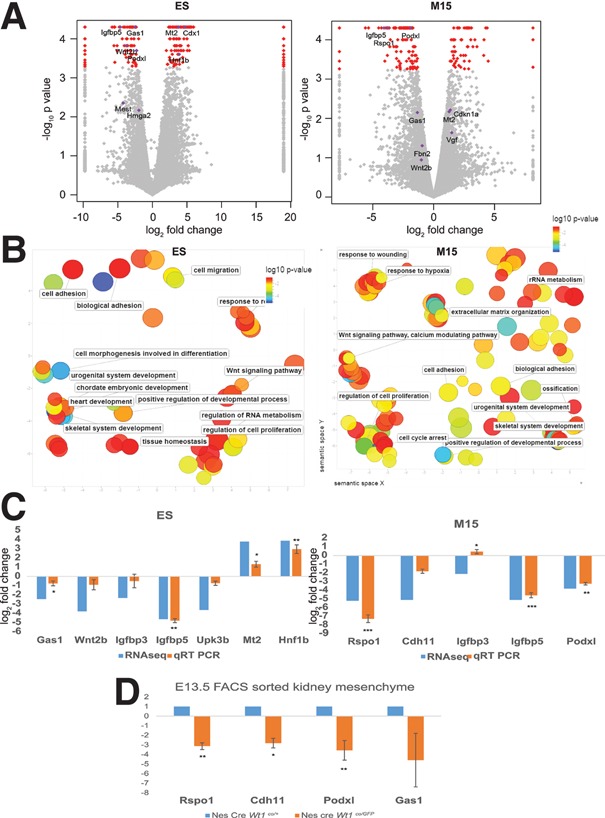
WT1 regulates the expression of a subset of the RNA-binding targets. (*A*) Volcano plot of transcriptome changes of E14 and *Wt1* knockout ES cell lines (*left*) ( *n* = 2) and *Wt1* stable knockdown in the M15 cell line compared with the *lacZ* controls (*right*) (*n* = 2). The *X*-axis is log_2_ fold change, and the *Y*-axis is log_10_ *P*-value. Selected genes are highlighted. (*B*) REVIGO plot of GO analysis based on *P*-values of the differentially regulated genes in ES (*left*) and M15 (*right*) upon *Wt1* knockout/knockdown, respectively. (*C*) WT1-interacting and regulated targets were validated by qRT–PCR. RNA changes in ES cells (*left*) and M15 cells (*right*) compared between the knockout/knockdown and control. Log_2_ fold changes observed in RNA sequencing (RNA-seq) (*n* = 2) and qRT–PCR (*n* = 3) are represented by blue and red bars, respectively. (***) *P* < 0.0001; (**) *P* < 0.001; (*) *P* < 0.01, unpaired *t*-test. (*D*) RNA changes in GFP^+^ FACS-sorted embryonic day 13.5 (E13.5) kidney cells compared with litter-matched cre control. *n* = 3.

As the WT1 RNA interaction data support a role in RNA stability, we assessed whether the expression levels of these targets were also regulated by WT1. The results by CuffDiff analysis (Trapnell et al. 2012) are summarized in Figure 4A. Of the differentially expressed genes in ES cells on *Wt1* knockout (using an adjusted *P*-value of 0.05 and with fragments per kilobase of transcript per million mapped reads [FPKM] ≥1 in at least one condition), 92 genes were up-regulated, and 67 genes were down-regulated. In M15, 156 genes were differentially expressed on *Wt1* knockdown (also see Supplemental Fig. S4), with 50 genes up-regulated and 106 genes down-regulated.

In M15 cells, 40% and 26% of differentially regulated genes were identified by RIP-seq and FLASH, respectively. In ES cells, 26% of differentially regulated genes were identified as interacting with WT1 by RIP-seq. Forty-five of the differentially regulated genes in M15 cells were found to have 267 gene self-hybrids from the FLASH analysis. Importantly, 252 of these hybrids were associated with down-regulated genes, and very few of the WT1-interacting RNAs (6%) were up-regulated. GO enrichment analysis of genes differentially regulated in both ES and M15 cells upon down-regulation of *Wt1* showed enrichment for pathways such as cell adhesion, positive regulation of developmental processes, wnt signaling, cell proliferation, skeletal system development, and urogenital system development (Fig. 4B; Supplemental Tables S6, S7). These processes are all disrupted through *Wt1* mutation or loss, but the mechanistic details have remained incomplete (Chau and Hastie 2012).

The expression changes found in the RNA sequencing (RNA-seq) data were validated by quantitative RT–PCR (qRT–PCR) in both ES cells and M15 cells for representative genes associated with the most significant GO terms. This analysis confirmed the down-regulation of the following RNA-binding targets with significant *P*-values. These include *Rspo1* and *Wnt2b* for wnt signaling, *Cdh11* and *Podxl* for adhesion, *Igfbps* for IGF signaling, and *Gas1* for proliferation. Other targets that followed a similar trend in both RNA-seq and qRT–PCR include *Wnt2b* and the mesothelial marker *Upk3b*. WT1 has been shown recently to regulate a subset of mesothelial origin progenitors toward the fat lineage (Chau et al. 2014). Selected up-regulated candidate genes, including *Mt2* and *Hnf1b*, were also validated by qRT–PCR (Fig. 4C).

In order to validate the findings in embryonic tissue, the expression of selected target genes (*Rspo1*, *Cdh11*, *Podxl*, and *Gas1*) was compared in FACS-sorted wild-type versus *Wt1* mutant metanephric mesenchyme from embryonic day 13.5 (E13.5) kidney cells. These were obtained by crossing the *Nes-Cre* and *Wt1*co alleles with a *Wt1GFP* knock-in model (Essafi et al. 2011). GFP^+^ cells from GFP control (*Wt1*^*co/GFP*^) and GFP^−^ cells from cre control (*Nes-Cre* ^*Wt1co*/+^) and *Wt1*-deficient (*Nes-Cre Wt1*^*co/GFP*^) were compared for expression. qRT–PCR analysis of the selected candidates showed up to 30-fold down-regulation of expression in the absence of *Wt1* (Fig. 4D), confirming the physiological relevance of these observations.

### The majority of WT1-dependent RNA-binding targets is not WT1 transcriptional targets

WT1 is known to both activate and repress transcription (for review, see Toska and Roberts 2014). In contrast, the global RNA interactome analysis supports a role for WT1 as an RNA-binding protein, functioning by interacting with the 3′ UTR of RNA and potentially regulating stability. Here, we showed that a significant number of the WT1-interacting RNA targets are reduced upon *Wt1* deletion/knockdown, reflecting WT1-mediated up-regulation at transcriptional and/or post-transcriptional steps.

**Figure 5.**
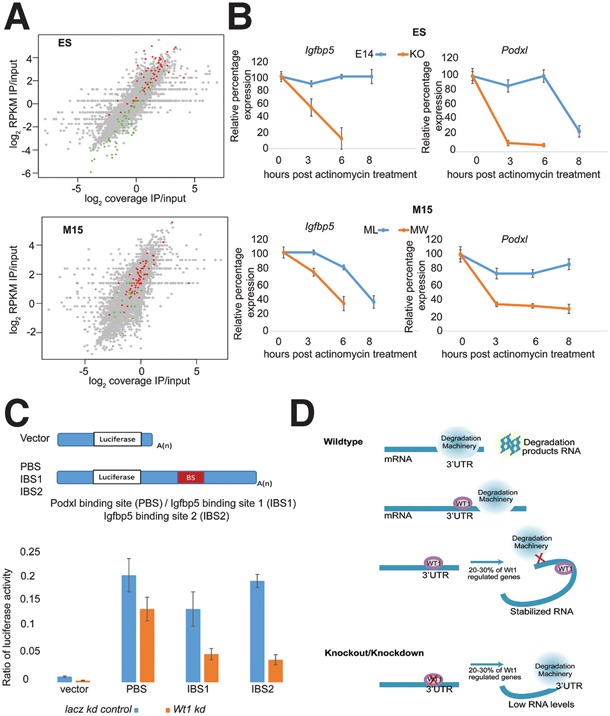
WT1 regulates RNA stability. (*A*) Scatter plots of log of the ratio of WT1 RIP-seq RPKM values to input (*Y-*axis) compared with the log of the ratio of coverage of genome-wide (*X*-axis) and the 3′ UTR (gray symbols), up-regulated genes (green), and down-regulated genes (red) (ES [*top*] and M15 [*bottom*]). (*B*) Relative percentage expression (*Y-*axis) of genes after actinomycin treatment in hours (*X*-axis) compared between knockout/knockdown and control cells (ES [*top*] and M15 [*bottom*]). (*C*) Luciferase reporter activity of WT1-interacting UTR-binding regions transfected in knockdown cells compared with control. Vector-alone transfections represent background luciferase activity without binding regions. (*D*) A working model for WT1 RNA interaction and its functional significance.

Consistent with post-transcriptional effects of WT1, a correlation was observed between the ratios of coverage (proportion of the length of the 3′ UTR with reads mapped) and reads per kilobase per million mapped reads (RPKM) between RNA interaction and transcriptome changes (Fig. 5A; Supplemental Fig. S5A). Down-regulated genes in ES cells have increased WT1 coverage and RPKM ratios in comparison with unregulated genes (*P* < 2.2 × 10^−16^ by Wilcoxon rank sum test) or up-regulated genes (*P* < 8.2 × 10^−14^ by Wilcoxon rank sum test).

Similarly, recurrent kmers occurring in 3′ UTRs were identified in the single-read FLASH data. The polyadenylation signal AATAAA had the highest *Z*-score, and three similar sequences were also found in the top 10 kmers. However, on correlation with down-regulation, the TGTAAAT motif was found by MEME (Bailey and Elkan 1994) at 294 sites (*E*-value 2.7 × 10^−562^), which is different from the motif identified by RIP-seq (Supplemental Fig. S5B,C).

To investigate whether down-regulated WT1 target mRNAs are also directly regulated by transcription, we compared them with published WT1 ChIP-seq (chromatin immunoprecipitation [ChIP] combined with high-throughput sequencing) data (Motamedi et al. 2014). On comparing the 156 differentially regulated genes from the M15 transcriptome analysis with the published set of 1771 WT1 ChIP-seq targets, there were 22 overlapping targets. Of the 38 most down-regulated and interacting RNA targets identified in our study, only seven were present in the above transcriptional target data set (Supplemental Table S8). A summary of the overlap between the RNA-interacting candidates, differentially regulated targets, and ChIP-seq targets identified by the different analyses in relation to the transcriptome analysis is shown in Supplemental Figure S5D.

### WT1 regulates RNA stability

To test whether, as hypothesized, WT1 regulates the turnover of target RNAs, wild-type and *Wt1*-depleted cells were treated with actinomycin, and RNA decay was measured using qPCR. As shown in Figure 5B, both *Podxl* and *Igfbp5* mRNAs decay more rapidly in ES and M15 cells when *Wt1* is deleted or down-regulated, respectively. To address whether WT1 is regulating gene expression through direct interactions with the 3′ UTR of mRNAs, fragments with WT1-binding sites were cloned into a luciferase reporter vector. Reduced luciferase activity was observed in *Wt1* knockdown cells transfected with WT1-interacting 3′ UTR fragments in comparison with the controls (Fig. 5C). Moreover, in a destabilized GFP reporter system, a rapid loss in GFP expression was observed in *Wt1* knockdown cells transfected with the *Igfbp5* UTR fragment that has the WT1-binding region in comparison with the control transfections, which showed no change in GFP expression. (Supplemental Fig. S5E). We conclude that direct WT1 binding to RNA enhances the levels of mRNA targets.

In summary, we present several lines of evidence to support the conclusion that WT1 regulates the stability of mRNAs through direct interactions. In addition to the direct experimental evidence, there are other considerations that support a role for WT1 in post-transcriptional regulation. Our data show that 94% of WT1-dependent interacting target RNAs were down-regulated, which deviates from the published WT1 transcriptional targets. Two studies have shown that 72% (Motamedi et al. 2014) and 77% (Dong et al. 2015) of WT1-dependent transcriptional targets are down-regulated, reflecting the fact that WT1 functions as a transcriptional activator or repressor. Consistent with this, only a small percentage of the down-regulated WT1-interacting RNAs overlap with the WT1 ChIP-seq targets (Supplemental Fig. S5D). Finally, the RNA-binding motifs identified become significant when ranking based only on down-regulated targets (Supplemental Fig. S5B,C). We propose that WT1 preferentially interacts with 3′ UTRs and that this interaction antagonizes binding of the RNA degradation machinery either directly or via stabilization of the mRNA structure, potentially through secondary structures (Fig. 5D). Although our analysis supports a role for WT1 in stabilizing RNAs, it does not exclude a role in promoting RNA turnover.

This study has revealed a few WT1-dependent genes—*Podxl* (Palmer et al. 2001) and *Rspo1* (Motamedi et al. 2014)—that are both transcriptional and post-transcriptional targets. Hence, for some genes, WT1 may chaperone them through various steps of the regulatory cascade. However, there is little overlap between the WT1-dependent target RNAs and WT1 transcriptional targets, emphasizing the key role of post-transcriptional processes in tissue development and homeostasis. The disparity in the number of WT1-interacting RNA and WT1-regulated RNAs may imply a role for WT1 in other post-transcriptional processes, including mRNA localization and translation. Recent genetic evidence linking Wilms’ tumor susceptibility with mutations in an exosome component (Astuti et al. 2012) and the miRNA processing genes (Wegert et al. 2015) supports the role of dysregulated RNA turnover and stability in the etiology of Wilms’ tumor. This resonates well with our demonstration that WT1 interacts with the 3′ UTR of RNA, influencing stability, thus arguing for a key regulatory role of WT1 RNA binding in the context of Wilms’ tumor.

## Materials and methods

### RIP-seq

WT1 RIP-seq on endogenous WT1 in ES and M15 cells was done by formaldehyde cross-linking followed by sonication. DNase-digested samples were immunoprecipitated with WT1 antibody. Interacting RNA was purified and processed for next-generation sequencing (NGS). The data obtained were analyzed using pyCRAC.

### FLASH

UV cross-linked M15 cells were immunoprecipitated with WT1-conjugated agarose antibodies. The extracts were treated with RNase and then formaldehyde cross-linked. The RNA protein complexes were purified after linker addition, separated on a gel, and transferred to a membrane. RNA was purified from the radioactive bands and processed for NGS analysis. Data analysis was done separately for the single reads and the hybrids obtained from FLASH.

### Transcriptome analysis by RNA-seq

RNA from mouse ES cell line E14, *Wt1* knockout ES line (KO1A), M15 control, a stable lentiviral *Wt1* knockdown M15 cell line, and a control *lacZ* lentiviral stable line was isolated using the Qiagen RNeasy minicolumns as per the manufacturer's protocol. The isolated total RNA (1 µg) was poly A-selected and subjected to library preparation with the NEBnext Ultra RNA library kit for Illumina sequencing.

### qRT–PCR validation

RNA was converted to cDNA and subjected to qPCR using SYBR Green for detection. Gene expression data were analyzed by the ΔΔCt method. The immunoprecipitation data were analyzed for fold enrichment. Students’ unpaired *t*-test was used for statistical validations.

### Analysis of gene expression changes in wild-type and mutant tissues

Crosses were set up with the *Wt1* floxed conditional with the *Wt1-GFP* knock-in Nestin Cre line, and E13.5 kidneys were single-cell-dissociated and FACS-sorted using the GFP signal. RNA was isolated from FACS-sorted control and mutant cells (identified by genotyping) using Trizol followed by cDNA synthesis and qRT–PCR.

### Endogenous RNA stability assay

Actinomycin-treated control and *Wt1* knockdown cells were analyzed for gene expression at different time points.

### Luciferase reporter assay

WT1-interacting 3′ UTR-binding fragments were analyzed for luciferase expression in a pIS1-based reporter assay in control and *Wt1* knockdown cells.

Detailed experimental procedures and data analysis are in the Supplemental Material.

## Supplementary Material

Supplemental Material
