# Supplementary material for: Transcription factor Wilms’ tumor 1 regulates developmental RNAs through 3′ UTR interaction
Source: Genes Dev. 2017 Feb 15;31(4):347–52. doi: 10.1101/gad.291500.116 (PMC5358755; doi:10.1101/gad.291500.116)
Supplement: Supplemental Material [file supp_31_4_347__index.html]

Transcription factor Wilms’ tumor 1 regulates developmental RNAs through 3′ UTR interaction — Supplemental Material 

# Transcription factor Wilms’ tumor 1 regulates developmental RNAs through 3′ UTR interaction

## Supplemental Material

- Supplemental\_Data.pdf
- Supplemental\_Table\_5\_miRNA\_sites\_Hybrids.xlsx
- Supplemental\_Table\_3\_M15\_FLASH\_GO.xlsx
- Supplemental\_Table\_7\_M15\_GO\_DAVID.xlsx
- Supplemental\_Table\_1\_ES\_RIPseq\_GO.xlsx
- Supplemental\_Table\_6\_ES\_GO\_DAVID.xlsx
- Supplemental\_Table\_4\_RIP\_and\_FLASH\_comparison.xlsx
- Supplemental\_Table\_8\_ChIPseq\_and\_Transcriptome\_comparison.xlsx
- Supplemental\_Table\_2\_M15\_RIPseq\_GO.xlsx
