# Supplementary material for: Transcription factor Wilms’ tumor 1 regulates developmental RNAs through 3′ UTR interaction
Source: Genes Dev. 2017 Feb 15;31(4):347–52. doi: 10.1101/gad.291500.116 (PMC5358755; doi:10.1101/gad.291500.116)

Transcription factor, Wilms' Tumour 1 regulates developmental RNAs through 3' UTR interaction.

Supplemental information

Materials and Methods

Cell lines: Mouse ES lines E14 and the *Wt1* KO ES cells were grown as monolayers and maintained on 0.1% gelatin coated dishes in GMEM media reconstituted with NEAA, Sodium pyruvate, 10% FCS, LIF and β-mercaptoethanol. For retinoic acid treatment, the above media without LIF was reconstituted with 1μM of Retinoic Acid in DMSO. Mesonephric cell line, M15 that expresses very high levels of WT1 was also used for the analysis. A stable lentiviral knockdown of *Wt1* expression in M15 cells (**Essafi et al., 2011**), which led to an approximate two to four-fold downregulation of *Wt1* expression was also used for the experiments, *lacZ* knockdown was used as a control. These cell lines were maintained in DMEM, 10% FCS. All cell lines were routinely tested for mycoplasma contamination.

RNA-IP analysis: Approximately 1×10^7 WT1 expressing cells were lysed in lysis buffer (50mM HEPES, 140mM NaCl, 1mM EDTA, 1% (v/v) Triton-X 100, 0.1% (w/v) sodium deoxycholate, protease inhibitor and 40U RNasin) on ice for 5 minutes followed by sonication using a 5sec on/off cycle for 2 minutes. To the sonicated extract, the following components were added, 25 mM $MgCl_2$, 5mM $CaCl_2$, RNasin (3μl of stock), RNase free DNaseI (6 μl of stock) and incubated at 37ºC for 15 minutes. The DNase digestion was stopped by addition of 20mM EDTA solution. Samples were centrifuged at 12,000 rpm for 5 minutes. Magnetic beads (Pierce) were bound with antibody of interest at a ratio of 20 μl of antibody with 4 μg of antibody (C19, SC192 X) at room temperature for 30 minutes. The magnetic beads were washed with the lysis buffer and used for the IP reactions, which were mixed with the sonicated, DNase digested extracts in 1 ml of lysis buffer with 0.5M EDTA and RNasin overnight at 4ºC. Following overnight IP, the samples were washed with the help of magnetic stand using 0.5 ml of buffer for each wash. Six washes with the lysis buffer and three washes with the wash buffer (50 mM HEPES, pH7.5, 500 mM NaCl, 1mM EDTA, 1% (v/v) Triton X 100, 0.1% (w/v) sodium deoxycholate, 40U RNasin per ml buffer) was carried out. Following the washes, samples were treated with proteinase K, incubated at 55ºC for 30 minutes and reverse cross linked (for formaldehyde cross linked samples) at 65ºC. Finally,

the samples were subjected to phenol-chloroform extraction and RNA precipitation with glycogen, Sodium acetate and absolute ethanol. The precipitated RNA was quantified by nanodrop and agilent bioanalyzer. Samples were processed either for next generation sequencing or qRT-PCR.

Next-generation sequencing: Double stranded cDNA synthesis of RNA-IP samples was performed as follows, the first strand cDNA synthesis was initiated using random primers, incubated at 70ºC for 10 minutes, followed by incubation on ice and reverse transcriptase reaction using AMV RT at 42ºC for 60 minutes, incubated on ice to terminate the reaction. This was followed by the second strand synthesis using the second strand enzyme and incubated at 16ºC for 2 hours, 10 µl of T4 DNA polymerase was added and incubated at 16ºC for 5 mins. The above reaction was terminated by the addition of 8.5µl of 0.2M EDTA, pH 8.0. Samples were then subjected to RNA digestion using RNase and proteinase K digestion, both at 37 ºC for 30 mins each followed by cleaning of the dsDNA product by phenol-chloroform extraction and precipitation with 5M ammonium acetate and 2.5 volumes of absolute ethanol. The samples were tested on bioanalyzer before being processed by the NEB next sequencing kit for illumina for NGS on Illumina platform.

FLASH:

UV Crosslinking was done on ice with $\lambda = 254$ nm in Stratalinker 1800 (Stratagene), at 400 mJ/cm^2.

Cell Lysis and WT1 bound complexes purification: Crosslinked cells were lysed by addition of ice-cold Lysis Buffer (20 mM Hepes pH 7.4, 150 mM NaCl, 0.4% NP-40, 2mM $MgCl_2$, 1 mM DTT, protease inhibitors (Roche, complete, EDTA-free), RNAse Inhibitor (Promega)), followed by addition of 10u of RQ1DNAse (Promega) to the samples and mixed by pipetting followed by incubation at room temperature for 10 min. Lysates were centrifuged at 14000rpm for 10 min at 4ºC and supernatant was collected.

Antibody preparation: Antibodies used were IgG conjugated protein A agarose beads and agarose beads conjugated with WT1 C19. The antibodies were washed with PBS/0.4% NP40 and incubated with the cell lysates for 60 min at 4°C. Supernatant was discarded, followed by beads washed twice with PBS-WB buffer (PBS reconstituted with 150mM NaCl, 2 mM $MgCl_2$, 0.4% NP-40), and once in 1xPBS buffer with 2mM $MgCl_2$.

RNAse treatment and formaldehyde crosslinking: Beads bound complexes were treated with 0.5 unit of RNaseA+T1 mix (RNace-IT, Stratagene) in 100 µl PBS with 2mM $MgCl_2$ buffer at 20°C for 10 min, followed by washes with PBS-WB buffer (PBS reconstituted with 150mM NaCl, 2 mM $MgCl_2$, 0.4% NP-40) twice, once with HS-PBS-WB (PBS reconstituted with 0.3 M NaCl, 2 mM $MgCl_2$, 0.4% NP-40), finally with 1xPBS.
Since this is an endogenous protein IP, formaldehyde crosslinking was done on the RNP complex bound beads using 0.5% formaldehyde in PBS for 3 min, followed by addition of 0.2 M glycine and 0.1 M Tris-HCl pH 8 and incubated for 5 min. Crosslinked complexes were subjected to denaturing washes (4 times) in urea buffer (20 mM Tris pH 7.4, 8 M UREA, 0.3 M NaCl, 0.4% NP-40) and additional incubation in urea buffer for 30 min at 4ºC.
Linkers' ligation and RNA-protein complexes recovery: WT1 RNP associated beads were subjected to washes with PNK buffer (50 mM Tris-HCl pH 7.5, 10 mM $MgCl_2$, 0.5% NP-40, 50 mM NaCl), four times. To remove unwanted 3'phosphate groups from bound RNA fragments the complexes were treated with TSAP phosphatase (Promega) using provided buffer for 40 min at room temperature. To inactivate the enzyme, the beads were washed twice with UB and four times with PNK buffer. This was followed by the 5' phosphorylation and radioactive labelling of RNA. The complexes on the beads were incubated with 40 units of T4 Polynucleotide kinase (New England Biolabs), initially with P^{32} labelled ATP for 45 min, followed by 1 mM cold ATP for 20 min, in PNK buffer with RNase inhibitors (RNasin, Promega) at room temperature. The reaction should provide 5' phosphates needed for downstream ligations. The beads were then washed as before, twice with urea buffer and four times with PNK buffer. WT1-bound RNA molecules were ligated together and with 3' linker (1 µM miRCat-33, IDT), overnight using 40 units of T4 RNA ligase 1 (New England Biolabs) in PNK buffer with RNase inhibitors at 16°C followed by washes as the previous step. Then using 40 units of RNA ligase 1, barcoded 5′ linkers (final conc. 5 µM; IDT, one for each sample) were ligated in RNA ligase 1 buffer with 1mM ATP for 3-6 hr at 20°C. The beads were washed as before with urea buffer and PNK buffer. Samples were boiled in NuPAGE protein sample buffer containing 100 mM Tris-HCl, 1%SDS, 100 mM β-mercaptoethanol for 3 min. The samples were cooled on ice for 5 min and centrifuged to recover the supernatant with RNA-protein complexes.
SDS-PAGE and Membrane Transfer: the above samples in NuPAGE SB plus SDS, ME (Life Technologies) were resolved on a 4%–12% Bis-Tris NuPAGE gel (Life Technologies) using NuPAGE SDS MOPS running buffer, transferred onto nitrocellulose membrane (GE Healthcare, Amersham Hybond ECL) in NuPage transfer buffer (Life Technologies) with

10% methanol for 1 hr at 100V. The membrane was exposed on film (Amersham) for 1 hr or overnight at -70ºC depending on the strength of the signal. The film was developed and aligned with the membrane to facilitate the isolation of radioactive bands corresponding to the WT1-RNA complexes.

Proteinase K Treatment and RNA Isolation: Excised bands were incubated with 150 μg of Proteinase K (Roche) in proteinase K buffer (50 mM Tris-HCl pH 7.8, 50 mM NaCl, 0.4% NP-40, 0.5% SDS, 5 mM EDTA) for 2 hr at 55°C. RNA was extracted with phenol-chloroform-isoamyl alcohol (PCI) mixture, pH 4.5 and ethanol precipitated overnight with 10 μg Glycogen (Ambion, Life Technologies).

cDNA Library preparation: The isolated RNA was dissolved in 12 μl of distilled RNAse-free water and reverse transcribed using miRCat-33 primer (IDT) with Superscript III Reverse Transcriptase (Life Technologies) for 1h at 50°C, followed by RNase H (New England Biolabs) treatment for 30 min at 37°C. cDNA was amplified using primers P5 and primer PE miRCat_PCR and TaKaRa LA Taq polymerase (Takara Bio). PCR products were separated on a 2% MetaPhor agarose (Lonza) gel with SYBRSafe (Life Technologies) in 1 x TBE at 4ºC. The gel band corresponding to 150-200bp was excised and the cDNA from this band was purified with MinElute Gel Extraction Kit (QIAGEN). The purified cDNA libraries were sequenced by NGS on Illumina platform.

qRT-PCR validation: Total RNA was isolated as described in the transcriptome analysis for gene expression analysis. Alternatively, for RNA interaction experiment validations, interacting RNA was precipitated as explained in the respective sections. The precipitated RNA was processed for cDNA synthesis instead of library preparation. cDNA synthesis was done by using the promega GOTaq qRT system using either oligodT or random primers for reverse transcription based on the experimental requirement. Most of the gene expression analysis studies were done using oligodT primers whereas the IP validations were carried out using the random primers. SyBR Green was used for detection. 18s rRNA was used as the control. Gene expression data was analysed by the del del Ct method. ES cells were compared to KO ES cells. For M15, since there was a 0.5 fold reduction of *Wt1* levels in lacZ knockdown in relation to the M15 control (**Supplemental Fig. S4**), for all calculations, lacZ and *Wt1* knockdown were compared. The IP data was analysed using the fold enrichment method. Students' unpaired t-test was used for statistical validations.

Motif Analysis: Integrating the kmer analysis with the transcriptome changes, the top 10 kmers (ranked by Z score) located in 3' UTRs were found to be associated with 892 genes, 19 of which were downregulated in ES whereas none in the upregulated category. In M15, the top 10 3' kmers occur in 1229 genes, 24 of which are downregulated and none upregulated. This sequence is similar to the PUM2 RNA binding motif TGTANATA (**Hafner et al. 2010**). These findings indicate that WT1 binds in the vicinity of the Poly (A) site and may either bind near Pum2 or have a comparable miRNA interaction mechanism to that proposed earlier (**Kedde et al. 2010**).

Hybrid database construction: The database used to search for hybrid reads was created by combining 3' UTR sequences, mature miRNA sequences and sequences for snRNA, snoRNA and rRNA (in detail, we used 1,915 miRNA sequences from mirBase; 40,341 3' UTRs; 863 snRNA; 949 snoRNA; and 222 rRNA from Ensembl 54, NCBIM37). Hybrid reads were those with two sub-sequences matching different database sequences, or matching the same database sequence with a discontinuity.

Gene Ontology Analysis and Representation: Gene Ontology terms were identified in different datasets by analyzing the genes for enrichment for significant processes using GOrilla (**Eden et al. 2009**) and visualizing the results using REVIGO (**Supek et al. 2011**). In some cases for an extended gene ontology search, DAVID (**Huang et al. 2009; 2009a**) functional analysis using the GO FAT category, was used.

Immunoprecipitation of endogenous WT1: Approximately $1x10^6$ WT1 expressing cells were lysed in RIPA buffer (50mM Tris, pH 8.0, 150 mM NaCl, 1% Triton X 100, 0.5% Sodium deoxycholate, 0.1% SDS, protease inhibitor cocktail) and the protein extract was quantified. The lysate was pre-cleared using protein A/G agarose beads for 1 hour at 4ºC. About 0.25 mg of the lysate was used per IP reaction with 2µg of antibody (agarose bound WT1 (SC-192) or agarose bound IgG (SC2345)). After overnight IP at 4ºC, the beads were washed with RIPA buffer (1ml x 4 times) and PBS (1 x 2 times). After the final PBS wash, the beads were resuspended in SDS loading buffer and processed for western blotting analysis. Most primary antibodies were left overnight at 4ºC at a dilution of 1:2500 and the secondary antibody was used at 1:10000 dilution for an hour at room temperature. The results were visualized with the help of the thermo scientific ECL kit. Antibodies used were as follows: WT1 (C-19, SC192/X), secondary antibody conformation specific anti rabbit HRP conjugate (CST L27A9).

RNA stability experiments: 5x10^4 Mouse ES and *Wt1* knockout ES cells as well as the M15 *lacZ* knockdown control and *Wt1* knockdown cells in 24 well plates were treated with actinomycin at 10µg/ml concentration. Cells were collected after 0, 3, 6 and 8 hours of treatment, resuspended in Trizol (100 µl), followed by addition of chloroform (20 µl) and vortexed. Samples were centrifuged at 13000 rpm for 15 minutes at 4ºC. Supernatant transferred to new 1.5ml tubes with 1µl of genelute. RNA was precipitated by addition of ethanol (70 µl), samples were vortexed and incubated at room temperature for 10 minutes, followed by centrifugation at 13000 rpm for 10 minutes at 4ºC. RNA pellets were washed with 70% ethanol and resuspended in 15 µl of RNase free water. RNA concentration was estimated by nanodrop and following oligodT primed cDNA synthesis, qRT-PCR was done as explained earlier using βactin as the reference gene.

Luciferase Reporter Assay: WT1 interacting hybrid sequences were separated to each half of the hybrid. This sequence was confirmed to be present both in the WT1 interacting single reads from FLASH dataset as well as the RIP-seq dataset. Primers were designed so as to include about 100bp flanking sequence to the WT1 interacting region referred to hereafter as binding site (BS). Each half of a hybrid thus amplified was denoted as BS1 and BS2. PCR amplified fragments were cloned into the pIS1 vector (Hmga2 3'UTR wt luciferase (Luc-wt) was a gift from David Bartel (Addgene plasmid # 14785) using the XbaI-NotI sites thus replacing the HMGA2 fragment with the binding sites, which were confirmed by restriction digestion confirmation and sequencing. pIS0 plasmid was used as the luciferase transfection control. 1x10^5 M15 *lacz* control knockdown and *Wt1* knockdown cells were transfected with 1µg each of the pIS0 transfection control plasmid and different versions of the binding site pIS1 based plasmids as indicated using 4 µg of lipofectamine. Following overnight transfection, media was changed, 30 hours post transfection, cells were collected and lysed with passive lysis buffer (500 µl). Luciferase measurements were done on a fluostar omega plate reader using the Dual Luciferase Reporter Assay reagents as per manufacturer's instructions. Briefly, 50 µl of the LAR II reagent was pipetted into wells. 20 µl of the lysate was added to these wells, mixed by pipetting and firefly luciferase (control) measurements were recorded. This was followed by addition of 50 µl of the Stop and Glo reagent, mixed by pipetting, followed by measurements of renilla luciferase (with the WT1 binding sites). Measurements were done on biological duplicates and technical triplicates. Background luminescence was detected based on measurements from the untransfected controls. Renilla activity to Firefly activity ratios were obtained as normalized to transfection efficiency.

Control vector with no binding site was used as a reference to compare the activity with the PBS1 (*Podxl* binding site 1) and IBS1/ IBS2 (*Igfbp5* binding site 1/2).

GFP Reporter Assay: Igfbp5 binding site 2 (IBS2) as explained above was PCR amplified and cloned into EcoRI/NotI sites of GFPd2 plasmid (pCAG-GFPd2 was a gift from Connie Cepko (Addgene plasmid # 14760) in tandem with the UTR, and confirmed by restriction digestion and sequencing. $1x10^5$ M15 *lacz* control knockdown and *Wt1* knockdown cells were transfected with 1µg of either GFPd2 vector alone or the IBS2-GFPd2 plasmid using 2.5 µg of lipofectamine. Following overnight transfection, media was changed. Cells were collected after 0, 3, 6 hours post media change and lysed with RIPA lysis buffer (0.1 ml). Protein concentrations were estimated and 20 µg whole cell lysates were electrophoresed on 10 % SDS PAGE. GFP reporter expression was assessed by immunoblotting with GFP antibody (TA150041). WT1 expression was confirmed with antibody against WT1 (ab 89901) and HSP90 antibody (BD biosciences, 610419) was used to assess equal loading.

References:

Eden E, Navon R, Steinfeld I, Lipson D, Yakhini, Z. 2009. "GOrilla: A Tool For Discovery And Visualization of Enriched GO Terms in Ranked Gene Lists", *BMC Bioinformatics* **10**: 48.

Hafner M, Landthaler M, Burger L, Khorshid M, Hausser J, Berninger P, Rothballer A, Ascano M Jr, Jungkamp AC, Munschauer M et al. 2010. Transcriptome-wide Identification of RNA-Binding Protein and MicroRNA Target Sites by PAR-CLIP. *Cell* **141**:129–141

Huang DW, Sherman BT, Lempicki RA. 2009. Systematic and integrative analysis of large gene lists using DAVID Bioinformatics Resources. *Nature Protoc*. **4**: 44-57.

Huang DW, Sherman BT, Lempicki RA. 2009a. Bioinformatics enrichment tools: paths toward the comprehensive functional analysis of large gene lists. *Nucleic Acids Res*. **37**: 1-13.

Kedde M, van Kouwenhove M, Zwart W, Oude Vrielink JA, Elkon R, Agami R. 2010. A Pumilio-induced RNA structure switch in p27-3' UTR controls miR-221 and miR-222 accessibility. *Nat. Cell Biol*. **12**: 1014–1020.

Matsuda T, Cepko CL. 2007. Controlled expression of transgenes introduced by in vivo electroporation. *Proc Natl Acad Sci U S A*. **104**:1027-1032.

Mayr C, Hemann MT, Bartel DP. 2007. Disrupting the pairing between let-7 and Hmga2 enhances oncogenic transformation. *Science*. **315**:1576-1579.

Supek F, Bošnjak M, Škunca N, Šmuc, T. 2011. REVIGO summarizes and visualizes long lists of Gene Ontology terms. *PLoS ONE* doi:10.1371/journal.pone.0021800.

Supplemental Figure S1 associated with Figure 1

A) Specificity of the WT1 antibody used for RNA interaction studies, Immunoprecipitated with the mentioned controls (Protein A/G beads, IgG) or WT1 and immunoblotted against WT1.

B) The reads obtained from the different RNA-IP experiments that were used for the analysis using the PyCRAC software are represented.

C) RNA IPs were performed and validated using primers spanning different regions for Dach1, Igfbp5 and Mest, as highlighted in blue. Enrichment across specific regions were observed.

D) REVIGO plots of gene ontology terms associated with top 1000 WT1 interacting mRNA identified in the ES cell line, WT1 RIP-seq analysis. Also refer to **Table S1** for the complete list

E) REVIGO plots of gene ontology terms associated with top 1000 WT1 interacting mRNA identified in the WT1 RIP-seq of M15 cell line analysis. Also refer to **Table S2** for the complete list.

Supplemental Figure S2 associated with Figure 2

The density of clusters identified by FLASH for WT1 IP (blue bars), compared to IgG IP (grey bars), for all protein coding regions and the density at 5' and 3' ends. Replicates are shown.

Supplemental Figure S3 associated with Figure 3

A) Energy associated with select gene-self hybrids in the 3' UTR of downregulated genes

B) Number of 3' UTR hybrids in candidate transcripts in WT1 FLASH (blue) compared to the hybrids in IgG control (grey) FLASH

C) Heatmap representation of *Igfbp3, Upk3b* and *Ctdsp2* RNA-RNA hybrids identified in M15 WT1 FLASH analysis, compared to PARIS analysis (**Lu et al. 2016**). Both the data were analysed by the hyb pipeline and data generated is presented as a plot.

D) Location of miRNA binding sites within the hybrids identified in a few representative targets identified in the WT1 FLASH analysis. Also refer to **Table S5** for a complete list.

Supplemental Figure S4 associated with Figure 4

Wt1 expression changes in M (M15 control cells), ML- M15 *Lacz* knockdown control) and MW (M15 *Wt1* knockdown). The numbers represent $\log_2$ fold change in expression, normalized to 18s rRNA and compared to M15 control.

Supplemental Figure S5 associated with Figure 5

A) The coverage of the RNA IPed over the input control (Panel I) in correlation with their expression (Panel II) depicts the overlap between the downregulated genes which are a part of the WT1 RNA interactome.

B) Motifs identified by k-mer analysis on targets identified in ES RIP-seq and M15 RIP-seq analysis. The MEME motif identified is also presented which shows a GC rich recognition motif.

C) The top 10 recurring kmers in 3' UTRs identified in WT1 FLASH single read data, ordered by Z score (top). Kmers reordered by enrichment for downregulated genes (bottom, ** $p<0.01$ * $p<0.05$).

D) Table showing overlap of M15 WT1 RIP-seq, M15 WT1 FLASH analysis (both single reads and hybrids) and E18.5 ChIP seq data from **Motamedi et al. 2014,** compared to the transcriptome changes identified in M15 cells upon *Wt1* knockdown.

E) Immunoblotting of WT1 binding site transfected GFP reporter cell lysates with monoclonal GFP antibody and hsp90 was used as a loading control. WT1 levels were detected by antibody against WT1. Time points indicate collection hours post media change after overnight transfection.

Supplemental Tables

Table S1: Gene ontology terms identified by GOrilla analysis of top 1000 WT1 interacting mRNA, identified in the ES cell line, WT1 RIP-seq dataset.

Table S2: Gene ontology terms identified by GOrilla analysis of top 1000 WT1 interacting mRNA, identified in the M15 cell line, WT1 RIP-seq dataset.

Table S3: Gene ontology terms identified by GOrilla analysis of top 2000 WT1 interacting RNA, identified in the M15 cell line, WT1 FLASH dataset.

Table S4: Comparison of WT1 FLASH analysis in M15 cells to WT1 RIP-seq in ES cells and WT1 RIP-seq in M15 cells. Sheet 1 provides a summary, Sheet 2 is the FLASH dataset analysis, Sheet 3 is the WT1 RIP-seq data from ES cells and Sheet 4 is the WT1 RIP-seq data from M15 cells.

Table S5: List of miRNA binding sites identified by TargetScan in the WT1 interacting RNA that form intramolecular hybrids at the 3' UTR. Sheet 1 is a list of target site and the number of hybrids with a defined miRNA binding site. Sheet 2 is the sequence information of the above. Sheet 3 represents the different miRNAs that can target these hybrids.

Table S6: Gene Ontology terms identified by DAVID analysis of transcriptome changes in the WT1 expressing ES cell line compared to *Wt1* knockout ES cells.

Table S7: Gene Ontology terms identified by DAVID analysis of transcriptome changes in the WT1 expressing M15 cell line, *lacZ* shRNA expressing M15 cell line and *Wt1* knockdown shRNA expressing M15 cell line.

Table S8: ChIP-seq and gene expression data integrated list from **Motamedi et al. 2014**, compared to the WT1 interacting hybrid forming targets that are downregulated in the absence of WT1 and differentially regulated targets in M15. Yellow highlighted genes represent those identified in the common overlap between ChIP-seq and ChIP on Chip data in **Motamedi et al. 2014**. Blue highlighted genes are those that were identified to overlap with the 1771 WT1 ChIPseq targets (**Motamedi et al. 2014**)

Oligo details

**β ACTIN FP**	AAAAACAAAGCCATGCCAAT
**β ACTIN RP**	GTCCACCTTCCAGCAGATGT
**IGFBP5 FP1**	CTGTGAGCTGGTCAAAGAGC
**IGFBP5 RP1**	TCTTTTCGTTGAGGCAAACC
**18S rRNA FP**	GTAACCCGTTGAACCCCATT
**18s rRNA RP**	CCATCCAATCGGTAGTAGCG
**Wt1 RT FP**	GCCTTCACCTTGCACTTCTC
**Wt1 RT RP**	GACCGTGCTGTATCCTTGGT
**GAS1 FP**	CAGCTACGCCTACAGCCAGT
**GAS1 RP**	CGTGTGGTTGAGCTGGATAA
**IGFBP3 FP**	GAGCAGTACCCGCTGAGG
**IGFBP3 RP**	CTTTCCACACTCCCAGCATT
**PODXL FP**	CAGCATCCAGTGTCACCATC
**PODXL RP**	TGCACTTTTTGTGCTCTTGG
**HNF1B FP**	GACACTCCTCCCATCCTCAA
**HNF1B RP**	CTCCCTCTGGGGGATATTGT
**WNT2B FP**	TGTGTCAACGCTACCCAGAC
**WNT2B RP**	ACCACTCCTGCTGACGAGAT
**RSPO1 FP**	ATTCTGCTGGAGAGGAACGA
**RSPO1 RP**	CTCCTGACACTTGGTGCAGA
**CDH11 FP**	ACATTCATGCCACCAAGACA
**CDH11 RP**	CAGGCACATTGGCATGATAG
**MT1 FP**	ACCCCAACTGCTCCTGCT
**MT1 RP**	CGCCTTTGCAGACACAGC
**PODXL FP**	TTTTGAAAACTCCTTGAGGAATG
**PODXL RP**	GCTGGTTGACATTCACAAACAG
**DACH1 LE FP**	CGAAGTCCTTCCTGGAGATG
**DACH1 LE RP**	CCAAAGTGGCTTCCTTTACG
**MEST LE FP**	GCTGGGGAAGTAGCTCAGTG
**MEST LE RP**	TCTTAGCAAGGGCCAACTGT
**IGFBP3 LE FP**	CACAGCGCTCCGTGTAGA
**IGFBP3 LE RP**	CTCACTGCCCTCACTCTGCT
**IGFBP4 LE FP**	CCCTATGCTCTGGGGTTGTA
**IGFBP4 LE RP**	GGCTTATCCTGTAGGGCACA
**IGFBP5 LE FP**	GGGACACATGGACAGAGCTT
**IGFBP5 LE RP**	ATCTCCAGGCTCCCTCTTTC
**WNT5A FP**	TCCAGGTGAAGGAGGAGGTA
**WNT5A RP**	AAACCCACAAAACCAGGCTG
**PDGFRA FP**	CGCTCCGGGTATCATCTTCT
**PDGFRA RP**	GAGATGCTACTGACGCGTTG
**PEG3 FP**	GGAGTGTTCTGTCCCCTACC
**PEG3 RP**	AGGGATGGGTTGATTTGGGT
**CDH11 FP1**	GAGAAGTCCACGGTGAACTT
**CDH11 RP1**	TGGGTTTAAGCTGGCTGGAT

**PODXL FP1**	GGGCAGGCTGGAAAAGAAAA
**PODXL RP1**	AGGCCTGTGAGTTTAGTCCC
**FP BS1 PODXL UTR**	GCTCTAGACTACTTGTGCCTGAGCCCAGAGCAG
**RP BS1 PODXL UTR**	ATAAGAATGCGGCCGCTCATGAGAGGTGGGATAC
**RP BS1 PODXL UTR RI**	GGAATTCTCATGAGAGGTGGGATACAACAAG
**FP BS1 IGFBP5 UTR**	GCTCTAGAACAAGTGAACTCAGAATGTGTGC
**RP BS1 IGFBP5 UTR**	ATAAGAATGCGGCCGCGATGCACCCTTGGGTGCTG
**FP BS2 IGFBP5 UTR**	GCTCTAGAAGCCTGATTTTGATCCAAGAGAAG
**RP BS2 IGFBP5 UTR**	ATAAGAATGCGGCCGCAGATGACTGCTCAAGATGCGGAG
**RP BS1 IGFBP5 UTR RI**	GGAATTCGATGCACCCTTGGGTGCTGGCAGTTAC
**RP BS2 IGFBP5 UTR RI**	GGAATTCAGATGACTGCTCAAGATGCGGAG

A

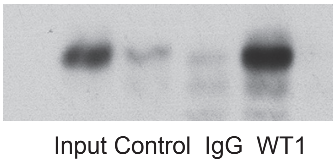

B

	E14_RNAIP	E14_RNAIPcon	M15_RNAIP	M15_RNAIPcon
Accepted hits	9619426	10274827	3154092	7362385
Filtered hits	8117025	9684491	2361179	6182562

C

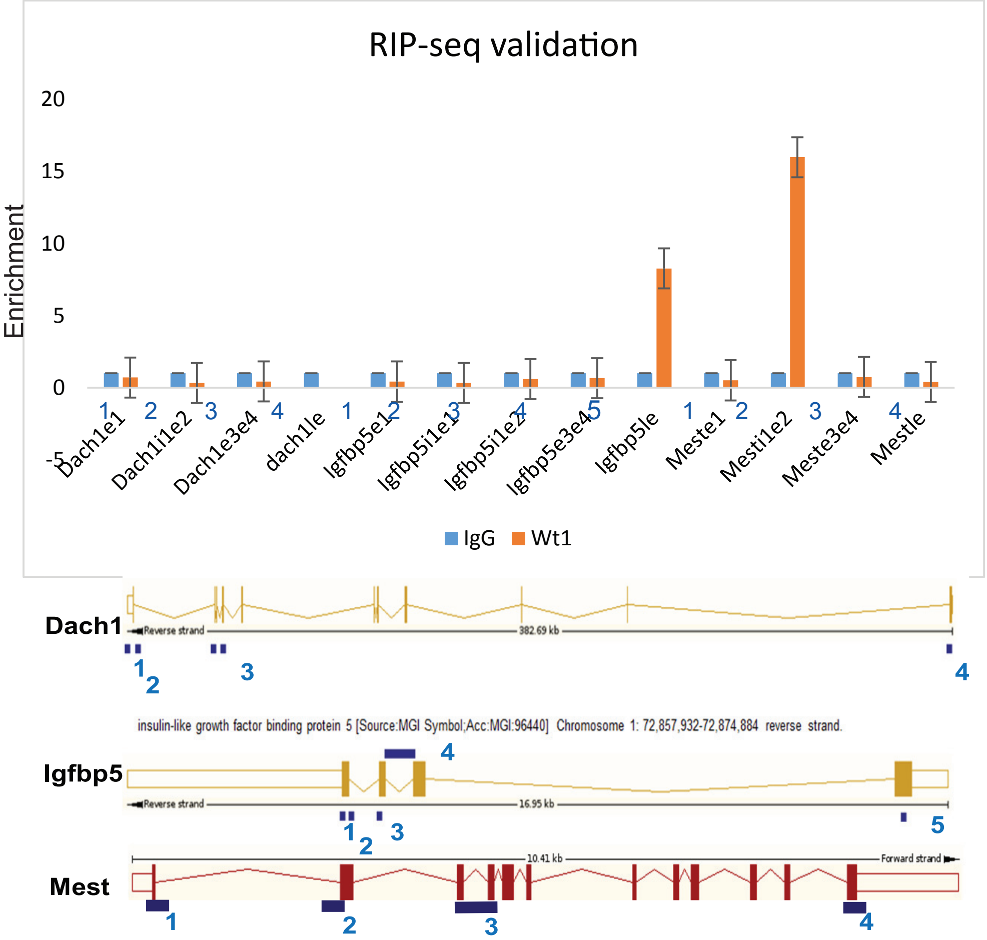

D

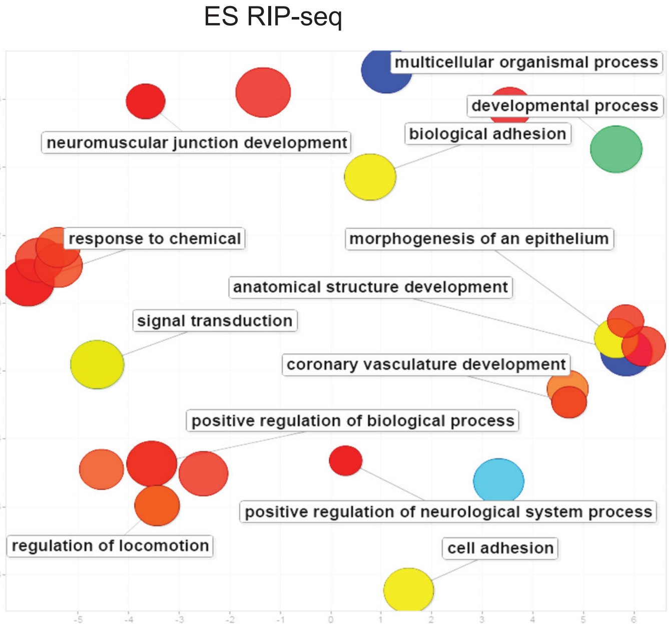

E

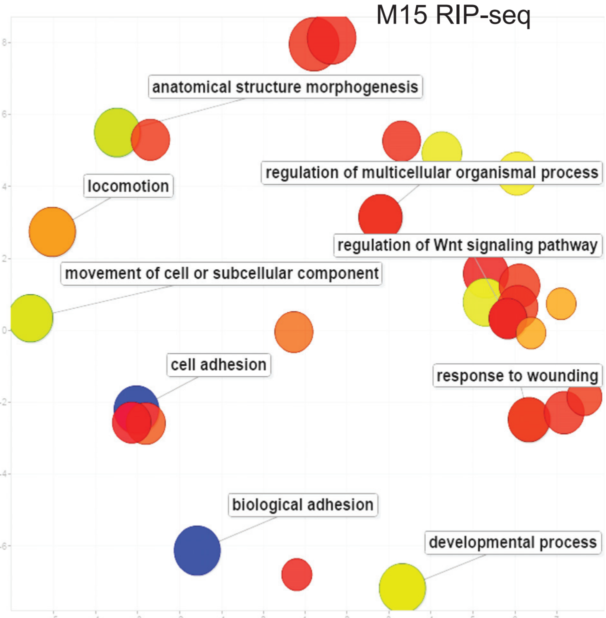

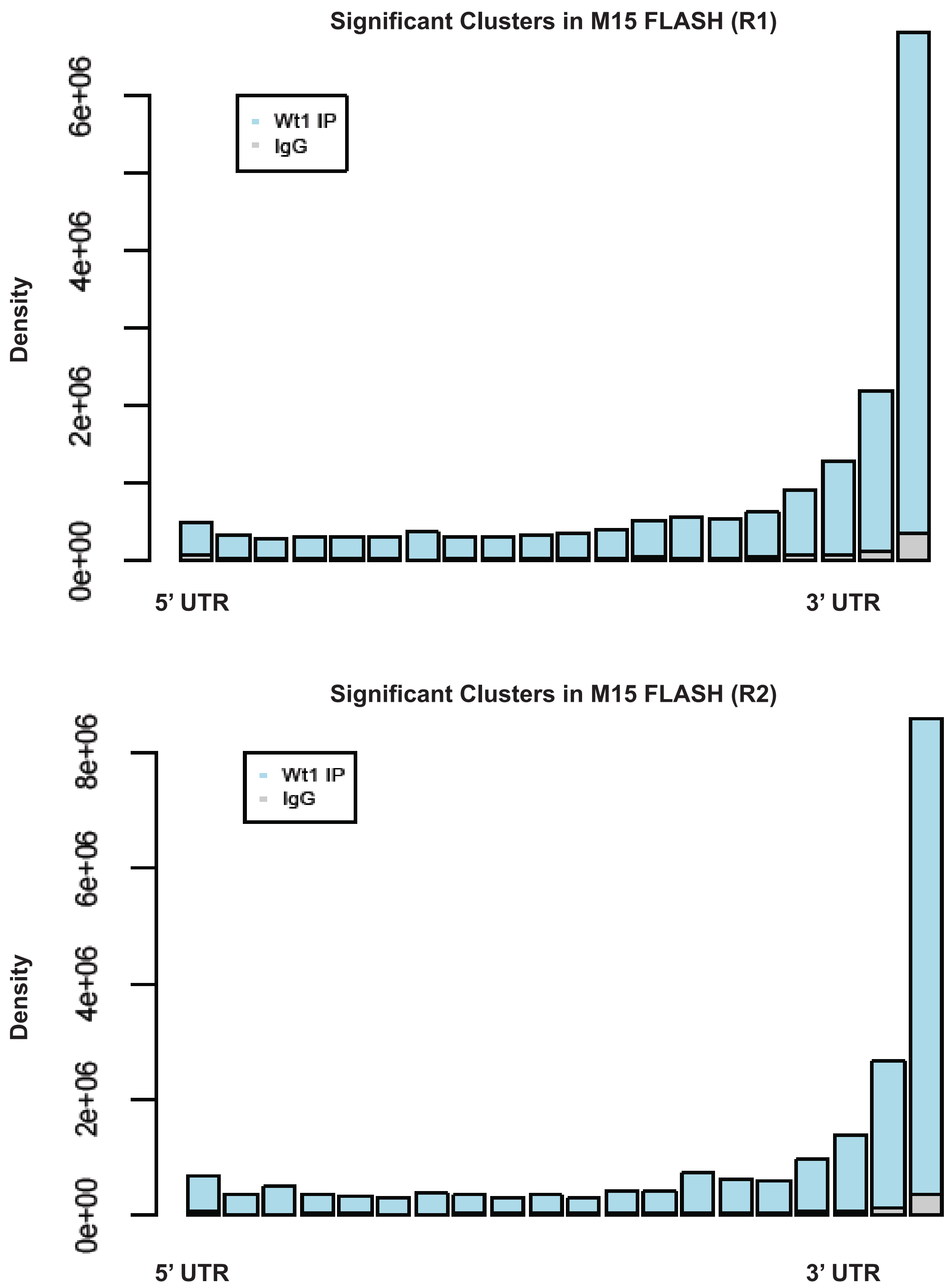
Significant Clusters in M15 FLASH (R1)
Wt1 IP
IgG
Density
0e+00
2e+06
4e+06
6e+06
5' UTR
3' UTR
Significant Clusters in M15 FLASH (R2)
Wt1 IP
IgG
Density
0e+00
2e+06
4e+06
6e+06
8e+06
5' UTR
3' UTR

Bharathavikru et al _FIGURE S3

A

Gene	Energy (no. hybrids)	Gene	Energy (no. hybrids)
Fmod	-25.2 (1)	D19Wsu162e	-19.9 (4)
Igfbp5	-21.6 (4)	Btg2	-17.8 (2)
Mgat3	-20.9 (4)	D0H4S114	-16.0 (1)

B

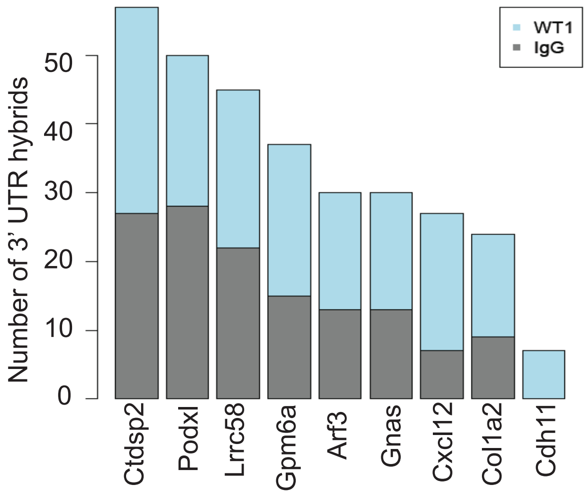

C

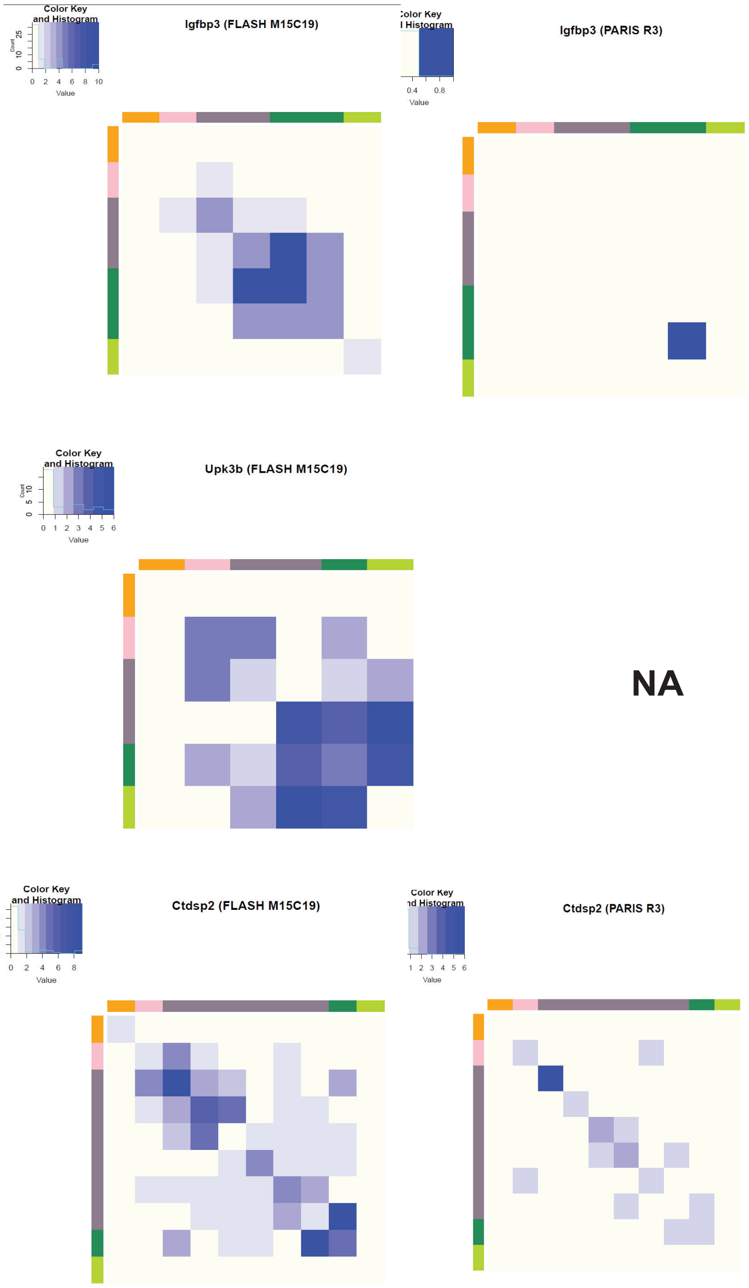

D

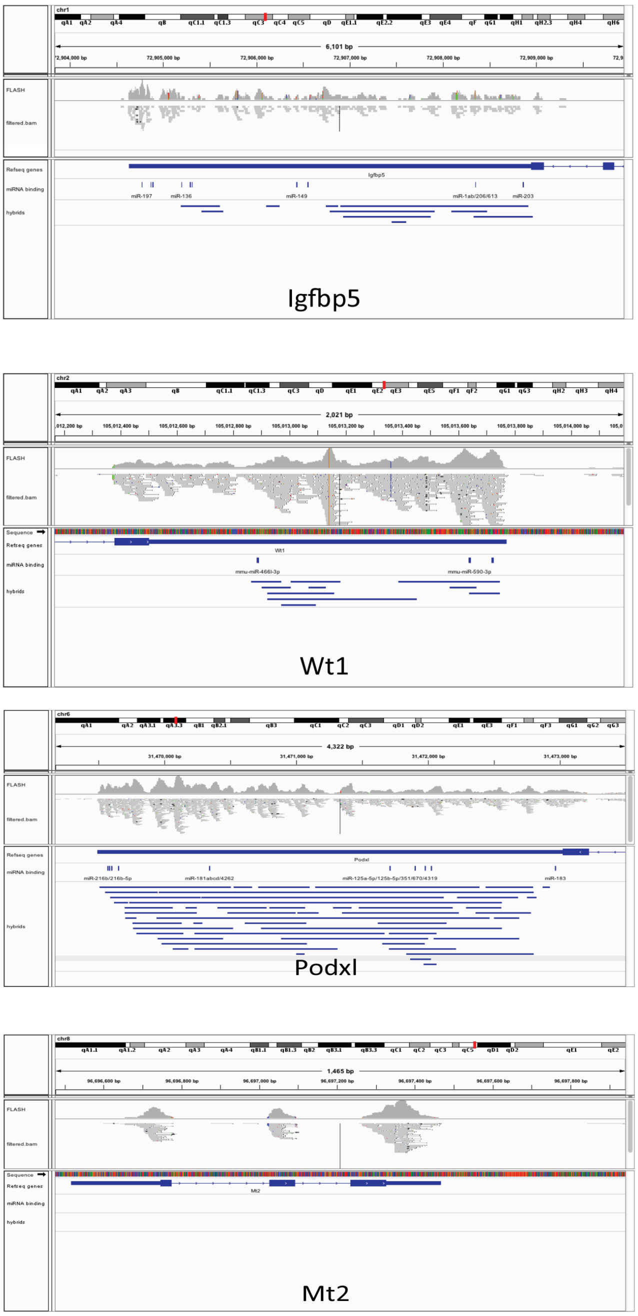

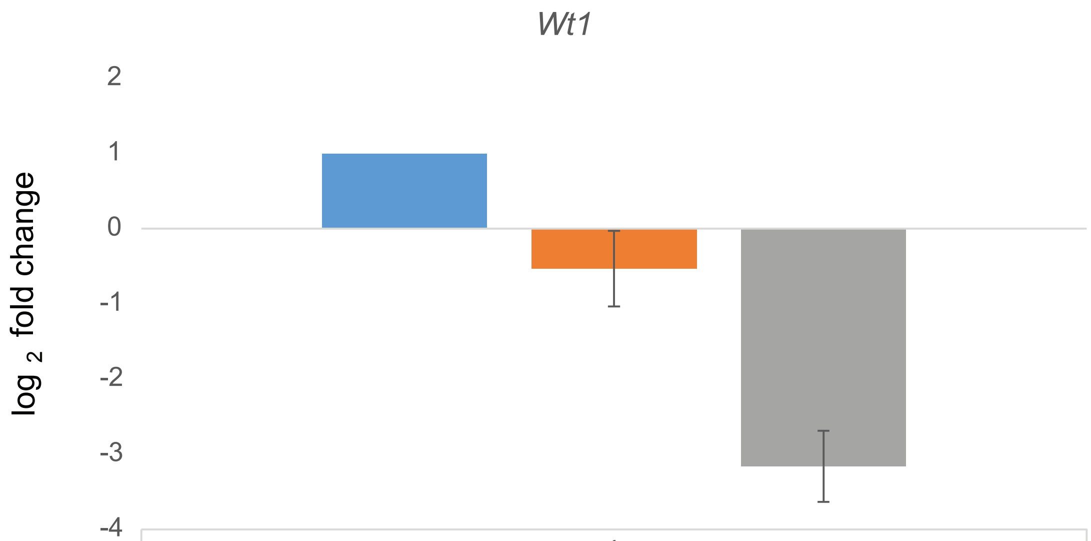

	1
M	1
ML	-0.53
MW	-3.16

Bharathavikru et al _FIGURE S5

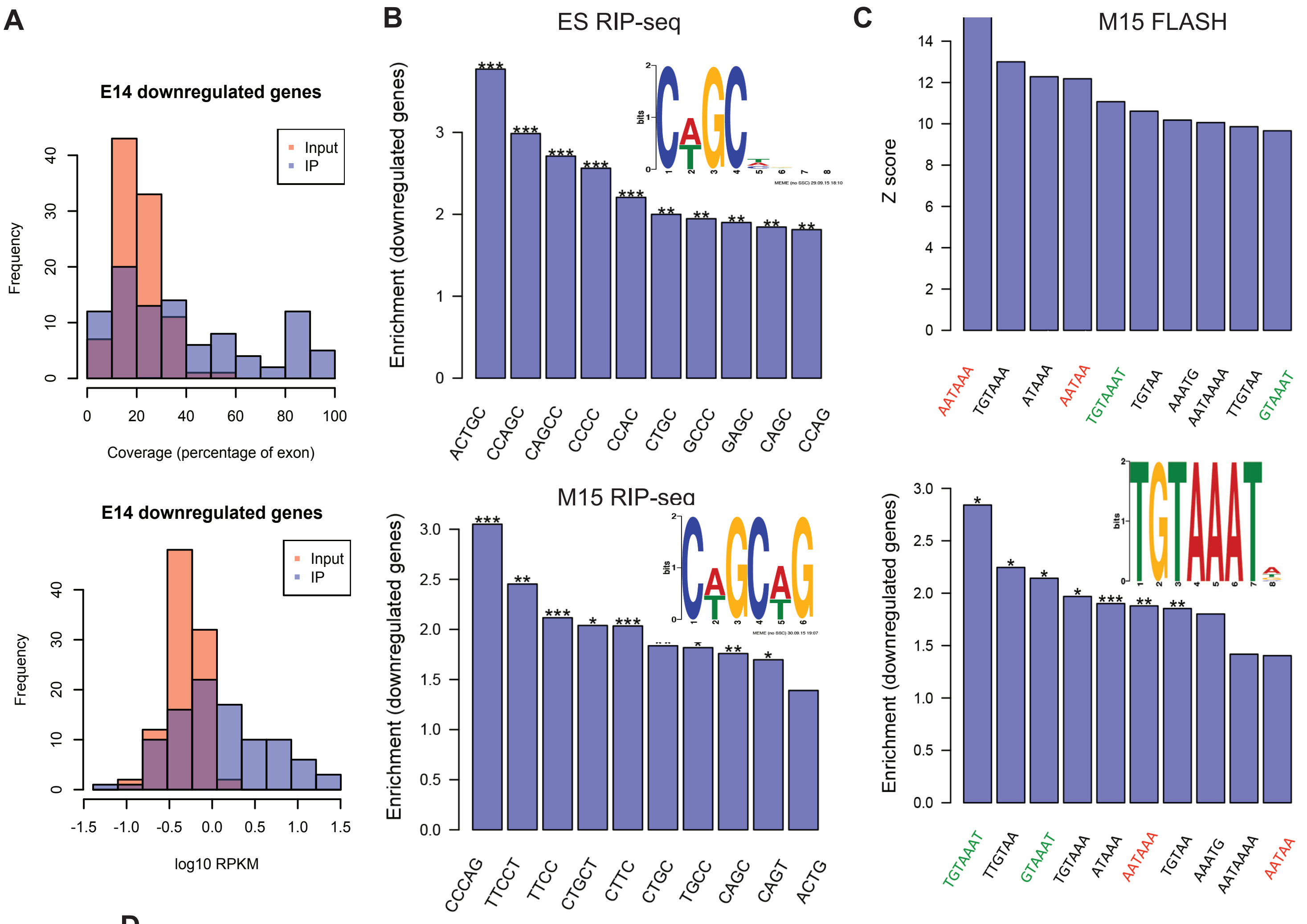

D

Dataset	Total Number of interacting mRNAs	Overlap with transcriptome (X=number of protein coding genes)	Percentage overlap with transcriptome %(X/156)	Overlap with downregulated transcriptome (X=number of protein coding genes)	Percentage overlap with downregulated transcriptome %(X/106)
M15 WT1 RIPseq	4968	62	40%	55	52%
M15 WT1 FLASH, single reads	4050	40	26%	34	32%
M15 WT1 FLASH, self hybrids	3955	45	29%	38	36%
E18.5 WT1 ChIP seq targets (**Motamedi et al., 2014**)	1771	22	14%	12	11%

E

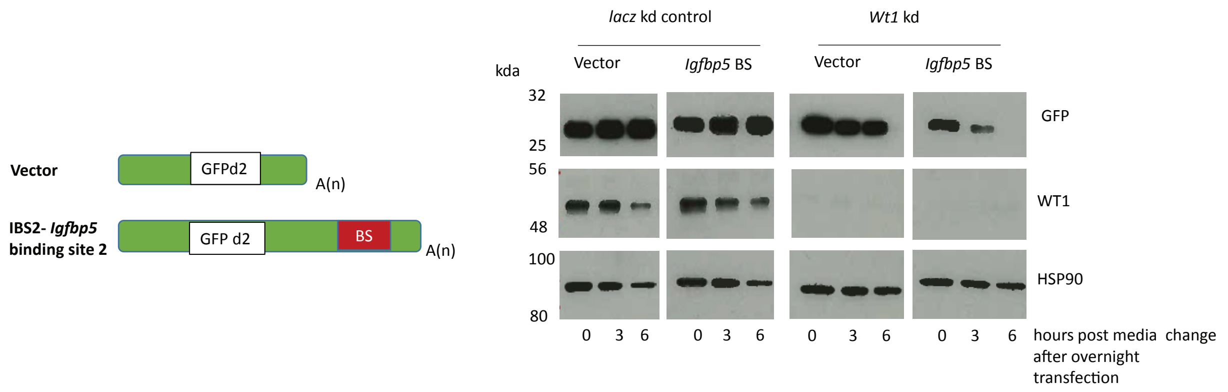

Supplement: Supplemental Material [file supp_gad.291500.116_Supplemental_Data.pdf]
